# Capturing alternative secondary structures of RNA by decomposition of base-pairing probabilities

**DOI:** 10.1186/s12859-018-2018-4

**Published:** 2018-02-19

**Authors:** Taichi Hagio, Shun Sakuraba, Junichi Iwakiri, Ryota Mori, Kiyoshi Asai

**Affiliations:** 1 Department of Computational Biology and Medical Sciences, Graduate School of Frontier Sciences, University of Tokyo, 5-1-5 Kashiwanoha, Kashiwa, 277-8561 Japan; 2 Artificial Intelligence Research Center (AIRC), National Institute of Advanced Industrial Science and Technology (AIST), 2-3-26 Aomi, Koto-ku, Tokyo, 136-0064 Japan; 3 Unique Co. Ltd., 6-6-20 Daita, Setagaya-ku, Tokyo, 155-0033 Japan

**Keywords:** RNA secondary structure, Dynamic programming, Base-pairing probability, Partition function

## Abstract

**Background:**

It is known that functional RNAs often switch their functions by forming different secondary structures. Popular tools for RNA secondary structures prediction, however, predict the single ‘best’ structures, and do not produce alternative structures. There are bioinformatics tools to predict suboptimal structures, but it is difficult to detect which alternative secondary structures are essential.

**Results:**

We proposed a new computational method to detect essential alternative secondary structures from RNA sequences by decomposing the base-pairing probability matrix. The decomposition is calculated by a newly implemented software tool, RintW, which efficiently computes the base-pairing probability distributions over the Hamming distance from arbitrary reference secondary structures. The proposed approach has been demonstrated on ROSE element RNA thermometer sequence and Lysine RNA ribo-switch, showing that the proposed approach captures conformational changes in secondary structures.

**Conclusions:**

We have shown that alternative secondary structures are captured by decomposing base-paring probabilities over Hamming distance. Source code is available from http://www.ncRNA.org/RintW.

## Background

Secondary structures of RNA are known to be thermodynamically fluctuated and the number of possible secondary structures of RNA are huge. We can predict the secondary structure by software tools, but on Boltzmann distribution of the secondary structure the probability of the ‘best’ secondary structure predicted is usually very small [1]. For example, the probability of the canonical ‘clover leaf’ secondary structure of a tRNAs is often less than one percent. One the other hand, the marginal probabilities on each base of the secondary structural contexts, such as base-pairs, loops, bulges, multi loops, are not necessarily very small and carry important structural information [2]. Among them, the **base-pairing probabilities (BPPs)** [3], which are often greater than eighty percent, are convenient to observe the local stability of the secondary structures. Furthermore, the estimators based on maximum expected accuracy, such as the MEA (Maximum Expected Accuracy) estimator [4] and the *γ*-centroid estimator [5], can be calculated by posterior decoding of BPPs without using further information of the RNA sequence [6].

Several functional RNAs, such as RNA thermometers and ribo-switches, change their functions by forming different secondary structures. It is difficult, however, to detect such structural changes. There are bioinformatics tools to predict suboptimal structures [7], but it is difficult to detect which alternative secondary structures are essential.

When the structural change occurs, the energy landscape, equivalently the probability distribution of the secondary structures, also changes, but we may expect that the information of the alternative secondary structures is included in the original probability distribution. For example, the two alternative structures of an aptamer verified by structure-specific RNase experiments were supported by two competing potential stems in the BPPs [8]. In order to characterize the alternative secondary structures more clearly, the other types of marginal probabilities, the distributions over Hamming distance are useful. The general idea has been proposed by [9] as an algorithm for exact calculation of distributions on integers applied to sequence alignment. For RNA secondary structures, algorithms and tools that calculate the probability distribution of the secondary structures over the Hamming distance from the reference structure has been proposed (RNAbor [10, 11], RintD [12]). If the distribution is concentrated near to the reference structure, the structure is regarded as reliable (i.e. low credibility limit). If there are multiple clusters of secondary structures, the distribution should have multiple peaks (See “Results” section). If the second reference structure is appropriately selected, we can obtain informative 2D distribution over Hamming distances from the two reference structures using RNA2Dfold [7] or RintD [12] (See “Results”section).

On the secondary structure over Hamming distance, a method to calculate approximate MEA estimators over Hamming distance has been introduced [13]. We propose a method to calculate exact decomposition of BPPs over each range of the Hamming distance and the exact MEA-based estimators (MEA estimator and *γ*-centroid estimator) by posterior decoding of decomposed BPPs as the representative secondary structure of the range of the Hamming distance. The existence of the cluster is visualized by the distribution over the Hamming distances from the two reference structures.

In order to establish the method to decompose the BPPs of a whole RNA sequence, we have developed RintW, a new computational tool that efficiently compute the exact base-pairing probability distribution over the Hamming distance from the reference structure. RintW is an extension of the calculation of partition function over Hamming distance in RintD [12]. RintD efficiently computes the secondary structure probability distribution over the Hamming distance from an arbitrary reference structure, by applying polynomial approach and Discrete Fourier Transform (DFT) to McCaskill’s algorithm [3]. In order to apply their approach to the base-pairing probabilities over Hamming distance, we derived outside algorithm for outside partition function from McCaskill’s algorithm of base-pairing probabilities [3]. RintW runs computational complexities of *O*(*L*^4^*H*_max_) in time and *O*(*L*^3^) in space, where *L* is the sequence length and *H*_max_ (<*L*) is the maximum values of the Hamming distance from an arbitrary structure.

## Methods

**Fig. 1:**
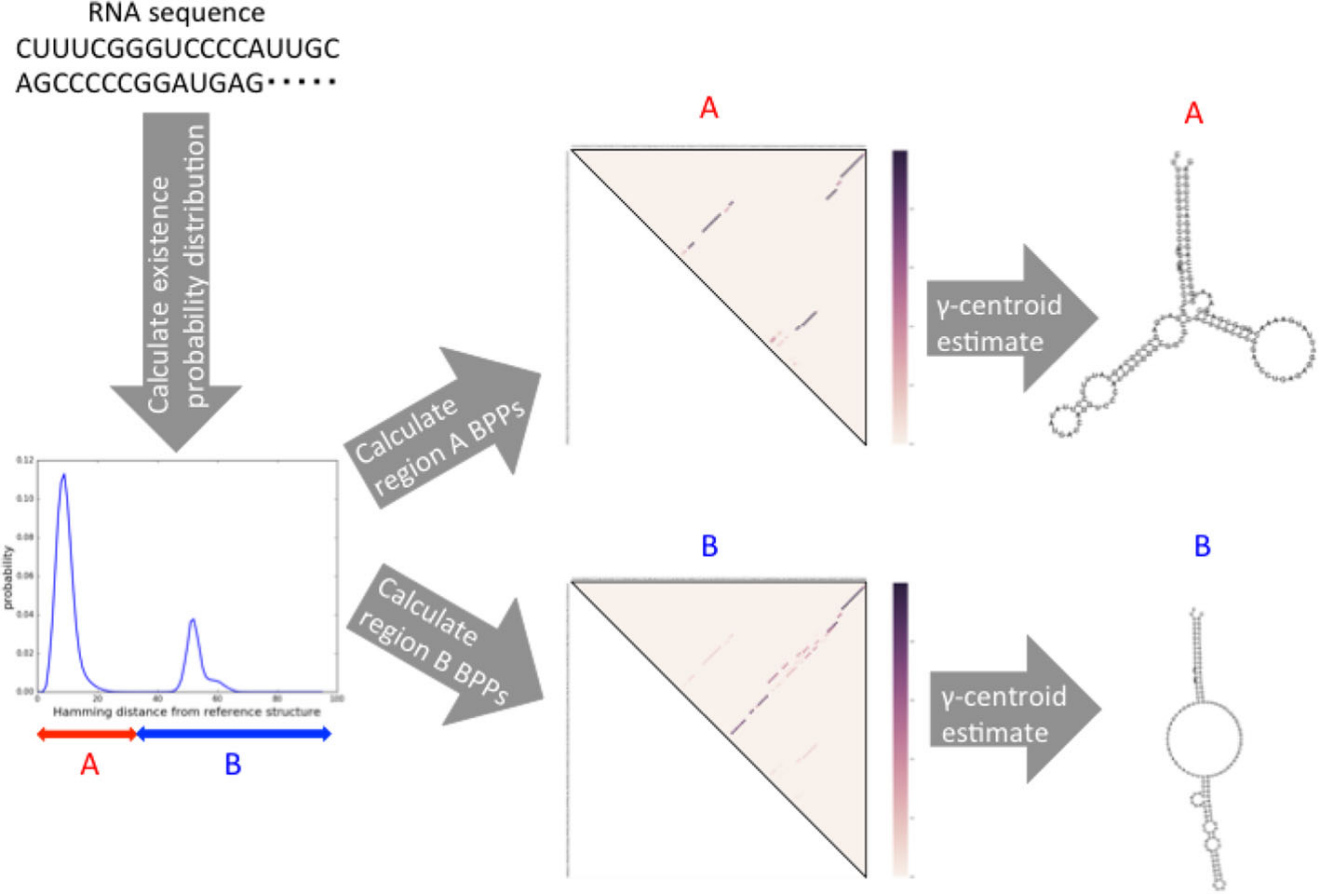
An overview of capturing the alternative secondary structures. The distribution of the secondary structures over the Hamming distance from the reference structure is calculated (left). The base-pairing probability matrix is calculated for each peak of the distribution (middle). The *γ*-centroid estimater of the secondary structure is calculated from each base-pairing probability matrix (right)

In this paper, we present a new computational method to detect the essential alternative structures from RNA sequences. Figure 1 shows an overview of the method. Firstly, we calculate an estimate of the secondary structure of the given RNA sequence, as the reference secondary structure. Secondly, the probability distribution of the secondary structure over the Hamming distance from the reference secondary structure is calculated by RintD [12] (Fig. 1 left). If there are multiple peaks in the distribution, which does not guarantee but implies that there are multiple clusters of secondary structures, we detect the ranges of Hamming distance for each potential cluster (Fig. 1 left, A and B). Thirdly, by newly implemented RintW, the base-paring probability matrix over each range of Hamming distance is calculated (Fig. 1 middle). Finally, the representative secondary structure for each cluster, which will be used as one of the reference structures for 2D analysis over Hamming distances, is estimated by posterior decoding of the corresponding base-pairing matrix (Fig. 1 right).

### 1D analysis over Hamming distance

#### Reference secondary structure selection

In the proposed method, the reference secondary structure is the structure estimated by CentroidFold [5]. Alternatively, of course, any reliable prediction by another tool, such as the minimum free energy structure by Mfold [14] can be used.

#### DP for secondary structure probability over Hamming distance

The distribution of secondary structures over Hamming distance from the reference strucure is calculated by RintD [12], while there are several tools for similar calculation (RNAbor [10, 11], RNA2Dfold [7]).

The Hamming distance between two secondary structures of an RNA sequence of the length *L* is defined as the Hamming distance of the upper triangle binary matrices *σ*_*i,j*_ as follows:

for 1≤*i*<*j*≤*L*,

1 $$ \begin{aligned} \sigma_{i,j} \,=\,\! \left[ \begin{array}{l} 1 \text{; } \text{a base-pair } (i,j) \text{ exists in the secondary structure}\\ 0 \text{; } \text{otherwise} \end{array}\right. \end{aligned}  $$

The probability distribution over Hamming distance, the marginal probability that the Hamming distance of the secondary structure is *d* from the reference structure *σ*, is written as follows:

2 $$\begin{array}{*{20}l} p(d,\sigma) = \frac{Z_{1,L}(d,\sigma)}{Z_{1,L}}, \end{array} $$

where *Z*_1,*L*_ is the partition function of all the secondary structures, and *Z*_1,*L*_(*d*,*σ*) is the partition function over Hamming distance *d* from the reference structure *σ*, which is the sum of all the Boltzmann factors of secondary structures whose Hamming distance from the reference structure *σ* is *d*. The partition function *Z*_1,*L*_ is calculated by McCaskill’s algorithm [3]. The partition function over Hamming distance, *Z*_1,*L*_(*d*,*σ*), can be calculated by a dynamic programming (DP) adding Hamming distance as an additional dimension of DP matrices to McCaskill’s algorithm. For practical implementation, however, the computational cost of this DP is too high. In the efficient calculation of RintD, this DP was mapped to a DP on polynomials and was converted to Discrete Fourier Transformation (DFT).

The DP on polynomials in RintD [12] is shown in Algorithm 1. In this algorithm, DP matrices, *Z*_*i,j*_, $Z^{1}_{i,j}$, $Z^{b}_{i,j}$, $Z^{m}_{i,j}$, $Z^{m1}_{i,j}$, are polynomials, whose terms store the sum of Boltzmann factors of subsequence [*i,j*] as the coefficients and the Hamming distance from the reference structure *σ* as the power of the dummy variable *x*. *Z*_*i,j*_ corresponds to the general case, $Z^{1}_{i,j}$ has exactly one outmost base pair whose 5^′^ base is *i*, $Z^{b}_{i,j}$ is the partition function conditioned by (*i,j*) base-pairing, $Z^{m}_{i,j}$ and $Z^{m1}_{i,j}$ correspond to the multi loops. The functions *f*_1_ to *f*_5_ are the free energy of unpaired bases depending on the structural contexts and the lengths of the subsequences.



The functions $g^{\mathrm {Z}}_{1}$ to $g^{\mathrm {Z}}_{8}$ are the gains of the Hamming distance for the transitions from the term in the right hand side of the equation to the left hand side *Z*’s, which mostly correspond to the number of the base-pairs in the reference structure where base-pair should not exist in the specific structural contexts. Using the following definitions

4 $$ \begin{aligned} g_{0}(i,j,h) &= \sum\limits_{p=i}^{h} \sum\limits_{q=h+1}^{j}\sigma_{p,q},\\ g^{\mathrm{Z}}_{0}(i,j) &= \sum\limits_{p=i}^{j-1}\sum\limits_{q=p+1}^{j}\sigma_{p,q}, \end{aligned}  $$

the gain functions are described as follows:

$$\begin{aligned} g_{1}^{\mathrm{Z}}(i,j) &= g^{\mathrm{Z}}_{0}(i,j), \\ g_{2}^{\mathrm{Z}}(i,j,h) &= g_{0}(i,j,h), \\ g_{3}^{\mathrm{Z}}(i,j,h) &= g_{0}(i,j,h+1) + g^{\mathrm{Z}}_{0}(h+1,j),\\ g_{4}^{\mathrm{Z}}(i,j) &= g^{\mathrm{Z}}_{0}(i,j) +1 - 2\sigma_{p,q}, \\ g_{5}^{\mathrm{Z}}(i,j,h,\ell) &= g^{\mathrm{Z}}_{0}(i,j) - g^{\mathrm{Z}}_{0}(h,\ell) +1 - 2\sigma_{p,q}, \\ \end{aligned} $$

$$\begin{aligned} g_{6}^{\mathrm{Z}}(i,j,h) &= g^{\mathrm{Z}}_{0}(i,j) - g^{\mathrm{Z}}_{0}(i+1,h-1) \\ &\quad- g^{\mathrm{Z}}_{0}(h,j-1) +1 - 2\sigma_{p,q}, \\ g_{7}^{\mathrm{Z}}(i,j,h) &= g^{\mathrm{Z}}_{0}(i,h-1) + g_{0}(i,j,h), \\ g_{8}^{\mathrm{Z}}(i,j,h) &= g_{0}(i,j,h-1). \end{aligned} $$

*Z*(*d*,*σ*), the partition function over the Hamming distance, is obtained as the coefficients of the polynomials as follows:

5 $$\begin{array}{*{20}l} Z_{1,L} = \sum\limits_{d=d_{\text{min}}}^{d_{\text{max}}} Z(d,\sigma)x^{d}, \end{array} $$

where *d*_max_ is the maximum Hamming distance from the reference structure, which is no greater than the length of the sequence, and *d*_min_ is the minimum, which is usually 0.

#### Accelerated calculation by discrete fourier transformation

Algorithm 1 includes the multiplication of the polynomials, which is computationally expensive. According to [9], distributed processing by DFT is available for dynamic programing of distribution on integer function. This acceleration has been implemented in RintD [12] for the partition function.

The DP on polynomials in Algorithm 1 can be converted to DP on complex numbers by substituting

6 $$\begin{array}{*{20}l} x = \exp\left[ 2\pi i \frac{r}{\Delta} \right], \end{array} $$

where *Δ*=*d*_max_−*d*_min_+1, then the DP matrices of the partition function over Hamming distance becomes matrices of complex numbers:

7 $$\begin{array}{*{20}l} \tilde{Z}_{i,j}(\sigma) = \sum\limits_{d={d_{\text{min}}}}^{{d_{\text{max}}}} Z_{i,j}(d,\sigma)\exp\left[ 2\pi i \frac{rd}{\Delta} \right]. \end{array} $$

In the DFT approach, the partition function over Hamming distance is rewritten as follows:

8 $$ \begin{aligned} Z(d,\sigma) &= \sum\limits_{s={d_{\text{min}}}}^{{d_{\text{max}}}} Z(s,\sigma) \delta_{sd} \\ &= \sum\limits_{s={d_{\text{min}}}}^{{d_{\text{max}}}} Z(s,\sigma) \sum\limits_{r={d_{\text{min}}}}^{{d_{\text{max}}}} \frac{\exp\left[2\pi i\frac{r(s-d)}{\Delta}\right]} {\Delta} \\ &= \frac{1}{\Delta}\sum\limits_{r={d_{\text{min}}}}^{{d_{\text{max}}}} \exp\left[2\pi i\frac{-rd}{\Delta}\right] \sum\limits_{s={d_{\text{min}}}}^{{d_{\text{max}}}} Z(s,\sigma)\exp\left[2\pi i\frac{rs}{\Delta}\right] \\ &= \frac{1}{\Delta}\sum\limits_{r={d_{\text{min}}}}^{{d_{\text{max}}}}\exp\left[2\pi i\frac{-rd}{\Delta}\right] \tilde{Z}_{1,L}(\sigma).  \end{aligned}  $$

In (8), $\tilde {Z}_{1,L}(\sigma)$ can be computed as a complex number by Algorithm 1, substituting *x*= exp[2*πi*(*r*/(*Δ*))] as shown in (7), and the entire (8) can be computed by DFT. Note that the above DP and DFT are computable in parallel.

#### Range detection

If the distribution over Hamming distance is concentrated around the reference structure, the structure is reliable (i.e. low credibility limit). If the distribution of Hamming distance has multiple peaks (as in Fig. 1 left), we detect the range of Hamming distance for each peak. It should be noted that a peak is generally not a guaranteed structural cluster but a potential candidate of a cluster, because the structures within a range of the Hamming distance from the reference structure may have mutual Hamming distance up to the double of the maximum Hamming distance of the range. On the contrary, a cluster whose members have small mutual Hamming distances is always observed as a peak in the distribution over Hamming distance. The first peak, however, tends to be a real cluster if the maximum Hamming distance of the range is reasonably small.

### Decomposition of base-pairing probability

#### DP for base-paring probabilities over Hamming distance

The base-pairing probability matrix over Hamming distance, $P^{b}_{ij}(d,\sigma)$, which is the marginal probability that the secondary structure has Hamming distance *d* from the reference structure *σ* and that the *i*-th base and the *j*-th base form a base-pair in the secondary structure, can be calculated by a DP algorithm adding Hamming distance as an additional dimension to the DP algorithm for base-pairing probability [3]. Because direct calculation of this DP is computationally impractical, we have developed a polynomial-DFT approach similar to RintD.

The original DP algorithm for base-pairing probabilities [3], however, included divisions of DP matrices, which is inappropriate for the polynomial-DFT approach. In order to avoid this problem, we rewrote the base-pairing probability over Hamming distance, as follows

9 $$ P^{b}_{ij}(d,\sigma) = \sum\limits_{t} \frac{Z^{b}_{ij}(d-t) W^{b}_{ij}(t)}{Z(d,\sigma)}.  $$

$Z^{b}_{ij}(d-t)$ and *Z*(*d*,*σ*) are obtained by Algorithm 1. The $Z^{b}_{ij}(d-t)$ is the inside partition function over Hamming distance, which is defined as the sum of all the Boltzmann factors of the structures of the subsequence [*i,j*] when (*i,j*) is a base-pair and the Hamming distance of subsequence [*i,j*] is *d*−*t*. *Z*(*d*,*σ*) is the partition function of the whole RNA sequence over Hamming distance, which is defined as the sum of all the Boltzmann factors of the structures of the whole sequence having Hamming distance *d*. $W^{b}_{ij}(t,\sigma)$ is the outside partition function over Hamming distance, which is defined as the sum of all the Boltzmann factors of the structures outside of the (*i,j*) base-pair when the Hamming distance outside of [*i,j*] is *t*. The algorithm for outside partition function is given in the next subsection.

The base-pairing probability matrix *P*^*b*^[*r*_min_,*r*_max_] on the range [*r*_min_,*r*_max_] of Hamming distance, is obtained by integrating each Boltzmann factor in (9) over the range of Hamming distance as follows:

10 $$ P^{b}_{ij}[r_{\min}, r_{\max}] = \frac{\sum_{d \in [r_{\min}, r_{\max}]} \sum_{t} Z_{ij}^{b}(d-t) W_{ij}^{b}(t)} {\sum_{d \in [r_{\min}, r_{\max}]} Z(d,\sigma)}.  $$

#### Outside partition function over Hamming distance

In order to calculate the base-pairing probability over Hamming distance by (9), we apply the polynomial-DFT approach to the outside partition function over Hamming distance. The dynamic programming on polynomials of the outside partition function is described in Algorithm 2. This algorithm corresponds to the outside algorithm of the stochastic context free grammar (SCFG), and iterations are computed from long (*i,j*) to short (*i,j*), while McCaskill’s algorithm of the partition function corresponds to the inside algorithm of SCFG and runs from short (*i,j*) to long (*i,j*).



In Algorithm 2, (11) represents the case that (*i,j*) base-pair is not included in any other base-pair, (12) represents the case of (*i,j*) base-pair being included in another base-pair (*h*,*ℓ*), while no base-pair in the subsequence (*h,i*) or (*j*,*ℓ*). In (13), (*i,j*) base-pair is included in another base-pair (*h*,*ℓ*), while at least one base-pair only in the subsequence (*h,i*) in the first line, at least one base-pair only in the subsequence (*j*,*ℓ*) in the second line, and at least one base-pair in both of the subsequences (*h,i*) and (*j*,*ℓ*) in the third line. This outside algorithm requires the partition functions *Z* and *Z*^*m*^ of Algorithm 1. *Z*_*i,j*_ is the partition function of subsequence [*i,j*], and $Z^{m}_{i,j}$ is the partition function of subsequence [*i,j*] that is a part of multi loop and that includes at least one base-pair. The functions *f*_2_ and *f*_4_ are the same as in Algorithm 1, the free energy of unpaired bases depending on the structural contexts.

**Fig. 2:**
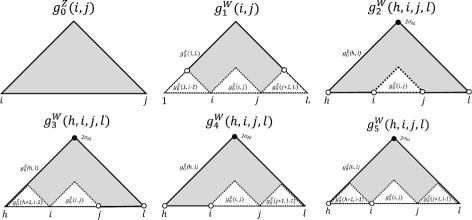
Schematic illustration of gain functions for the powers of polynomials in Algorithm 2

The functions $g^{\mathrm {W}}_{1}$ to $g^{\mathrm {W}}_{5}$ are the gain function of Hamming distance, for the transitions from the term in the right hand side of the equation to the left hand side *W*’s, which mostly correspond to the number of the paired bases in the reference structure where no base pair may exist in the specific structural contexts. The functions $g^{W}_{1}$ to $g^{W}_{5}$ are defined using $g^{\mathrm {Z}}_{0}$ in (4) as follows, and illustrated in Fig. 2.

$${\begin{aligned} g^{\mathrm{W}}_{1}(i,j) &= g^{\mathrm{Z}}_{0}(1,L) - g^{\mathrm{Z}}_{0}(i,j) - g^{\mathrm{Z}}_{0}(1,i-1) \\&\quad- g^{\mathrm{Z}}_{0}(j+1,L), \\ g^{\mathrm{W}}_{2}(h,i,j,\ell) &= g^{\mathrm{Z}}_{0}(h,\ell) - g^{\mathrm{Z}}_{0}(i,j) +1 - 2\sigma_{h,\ell}, \\ g^{\mathrm{W}}_{3}(h,i,j,\ell) &= g^{\mathrm{W}}_{2}(h,i,j,\ell) - g^{\mathrm{Z}}_{0}(h+1,i-1), \\ g^{\mathrm{W}}_{4}(h,i,j,\ell) &= g^{\mathrm{W}}_{2}(h,i,j,\ell) - g^{\mathrm{Z}}_{0}(j+1,\ell-1), \\ g^{\mathrm{W}}_{5}(h,i,j,\ell) &= g^{\mathrm{W}}_{2}(h,i,j,\ell) - g^{\mathrm{Z}}_{0}(h+1,i-1)\\&\quad - g^{\mathrm{Z}}_{0}(j+1,\ell-1). \end{aligned}} $$

$W^{b}_{ij}(d,\sigma)$, the outside partition function over Hamming distance, which is the sum of all the Boltzmann factors outside of the (*i,j*) base-pair, whose Hamming distance outside of (*i,j*) from the reference structure *σ* is *d*, is obtained as the coefficients of the polynomials as

14 $$ W^{b}_{i,j}(\sigma) = \sum\limits_{d} W^{b}_{ij}(d,\sigma)x^{d}.  $$

#### Accelerated calculation by discrete fourier transformation

Algorithm 2 also includes the multiplication of the polynomials, in the third term of (13). We applied DFT approach to the outside partition function over Hamming distance. Substituting same complex number to *x* as in (6), we obtain

15 $$\begin{array}{*{20}l} \tilde{W}^{b}_{i,j}(\sigma) = \sum\limits_{d={d_{\text{min}}}}^{{d_{\text{max}}}} W^{b}_{i,j}(d,\sigma)\exp\left[ 2\pi i \frac{rd}{\Delta} \right]. \end{array} $$

The outside partition function over Hamming distance is rewritten as

16 $$ \begin{aligned} W^{b}(d) &= \sum\limits_{s={d_{\text{min}}}}^{{d_{\text{max}}}} W^{b}(s) \delta_{sd} \\ &= \sum\limits_{s={d_{\text{min}}}}^{{d_{\text{max}}}} W^{b}(s) \sum\limits_{r={d_{\text{min}}}}^{{d_{\text{max}}}} \frac{\exp\left[2\pi i\frac{r(s-d)}{\Delta}\right]} {\Delta} \\ &= \frac{1}{\Delta}\sum\limits_{r={d_{\text{min}}}}^{{d_{\text{max}}}} \exp\left[2\pi i\frac{-rd}{\Delta}\right] \sum\limits_{s={d_{\text{min}}}}^{{d_{\text{max}}}} W^{b}(s)\exp\left[2\pi i\frac{rs}{\Delta}\right] \\ &= \frac{1}{\Delta}\sum\limits_{r={d_{\text{min}}}}^{{d_{\text{max}}}} \exp\left[2\pi i\frac{-rd}{\Delta}\right] \tilde{W}_{1,L}(\sigma).  \end{aligned}  $$

In (16), $\tilde {W}_{1,L}(\sigma)$ can be computed by Algorithm 2, substituting *x*= exp[2*πi*(*r*/(*Δ*))]. The entire (16) can be computed by DFT. Note that the above dynamic programing and DFT are computable in parallel.

### Secondary structure predictions using decomposed base-pairing probabilities

Once we obtain the partition functions over Hamming distance, *Z*^*b*^ and *W*^*b*^, we can calculate the base-pairing probabilities (BPPs) *P*^*b*^(*d*) on each Hamming distance *d* by Eq. (9), and then BPPs, $P^{b}_{ij}\left [r_{\min },r_{\max }\right ]$ for each peak by Eq. (10). As the representative secondary structures of each peaks, the *γ*-centroid estimator is computed from each corresponding BPP by the posterior decoding [4].

## Results

### Application to Lysine riboswitch

**Fig. 3:**
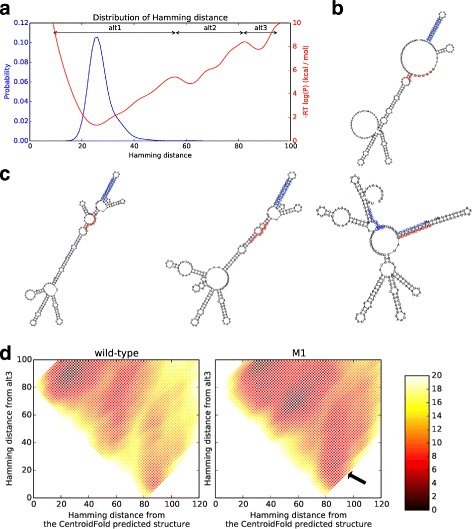
**a** The distribution of Lysine riboswitch secondary structures projected to the Hamming distance from the reference structure. Both the probabilities and their logarithm are plotted. **b** The secondary structure representations of alternative structures. **c** The alternative structures of the the Hamming distance range [9,56] (left), [56,82] (middle) and [82,95] (right, “alt3”). Terminator and antiterminator sequences are marked with circles (see the main text.) Figures were generated by RNAplot in the ViennaRNA package [7]. **d** The 2D distribution of Lysine riboswitch secondary structures projected to the Hamming distances from the structure predicted by CentroidFold and the alt3 structures, calculated by using RintD [12]. The probabilities were converted to the free energy (i.e. −*RT* log*P*) and were plotted. (left) The case with the wild-type RNA sequence. (right) The case with the M1 mutant. Secondary structures that have low Hamming distances to the alt3 structure are more stable with the M1 mutant than those with the wild-type (marked with an arrow). Free energies greater than 20 kcal/mol are plotted in white. Note the checkered pattern appears due to difficulties in achieving some Hamming distance constraints

We applied our method to an RNA called the Lysine riboswitch. The Lysine riboswitch RNA is 5’-UTR region of *lysC* and is known to be regulated by concentration of lysine [15]. The sequence was taken from *lysC* of *B. Subtilis* (J03294.1:2297–2537). The secondary structure predicted by CentroidFold (with *γ*=1) was chosen as a reference structure. First we analyzed the distribution of the structures over Hamming distance from the reference structure *σ*, represented as *P*(*d*,*σ*) in (2). Figure 3a represents the plot of *P*(*d*), where multiple peaks were observed in the distribution. We split the range of the Hamming distance at *d*=*d*^′^ such that *P*(*d*^′^−1)>*P*(*d*^′^) and *P*(*d*^′^)<*P*(*d*^′^+1). Furthermore, only the region *P*(*d*)> exp(*Q*/*RT*) was considered, where *Q*=−10 kcal/mol, *R* is the gas constant, and *T*=310 K (37 °C). As a result, ranges of [*r*_min_,*r*_max_] were determined to be [9, 56], [56, 82], and [82, 95]. The base-pairing probability matrices were then calculated according to the procedures described in “Methods” section. The *γ*-centroid estimator (*γ*=1) were then reconstructed by posterior decoding of the base-pairing probability matrices as the representative structures (alternative structures hereafter).

Figure 3b and c represent the secondary structure of the reference structure, and those of the alternative structures, respectively. As expected from the large Hamming distance change, the alternative structure obtained from the Hamming distance range [82,95] had a considerable structural change from the reference structure (hereafter we call this structure *alt3*). Furthermore, it can be seen that two experimentally important sequences (red and skyblue circles) of the RNA, form an antiterminator stem. Experimentally, it has been considered that 3’ pair of the antiterminator stem (colored skyblue in Fig. 3b also forms a terminator hairpin (skyblue and blue circles) and its transition is modulated by the concentration of lysine. Disrupting either of the antiterminator stem, or the terminator hairpin formation by mutations leads to the loss of the riboswitch function [15]. We further applied the RintD [12] algorithm to both the wild-type and a mutant *M1* that has modified sequence at the 5’-pair of the terminator hairpin, with mutations U234A and G235C. These mutations have been known to disrupt the pairing of the terminator hairpin. We use the structure predicted by CentroidFold, and alt3 as the reference structures for the RintD-2D [12]. Figure 3d shows the mutations to the RNA sequence increase the ratio of the alternative structure found in the wild-type sequence. It is thus inferred that a structural transition between the structure predicted by CentroidFold and alt3 reflects the functionally relevant structural change of the Lysine riboswitch.

We note that in the case of Fig. 3 the probability that a randomly sampled sequence falls into the Hamming-distance range of [82,95] is 1.43×10^−5^. These structures have a low total probability when RNA is not interacting to other molecules, but this weak peak may imply a potential structural change under the interaction with the ligand. We also note that such a low-probability structural cluster is hard to find using existing methods, such as the random sampling of the secondary structures.

### Application to ROSE element thermometer

The ROSE element thermometer is a functional RNA encoded in 5’-UTR of a mRNA, which changes its structure according to the temperature to regulate the translation of the downstream mRNA. The ROSE element thermometer prohibits the binding of ribosomes to the Shine-Dalgano (SD) sequence in 30 °C, but the structure change in 45 °C enables ribosomes to bind SD sequence to promote the translation of the mRNA.

**Fig. 4:**
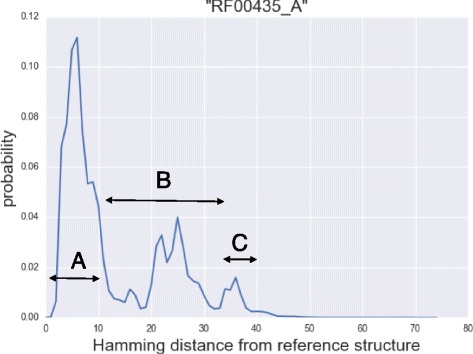
Distribution over Hamming distance (1D) of ROSE element thermometer. The reference structure was taken from the *γ*-centroid estimator (*γ* = 1) with the temperature of 30 °C

**Fig. 5:**
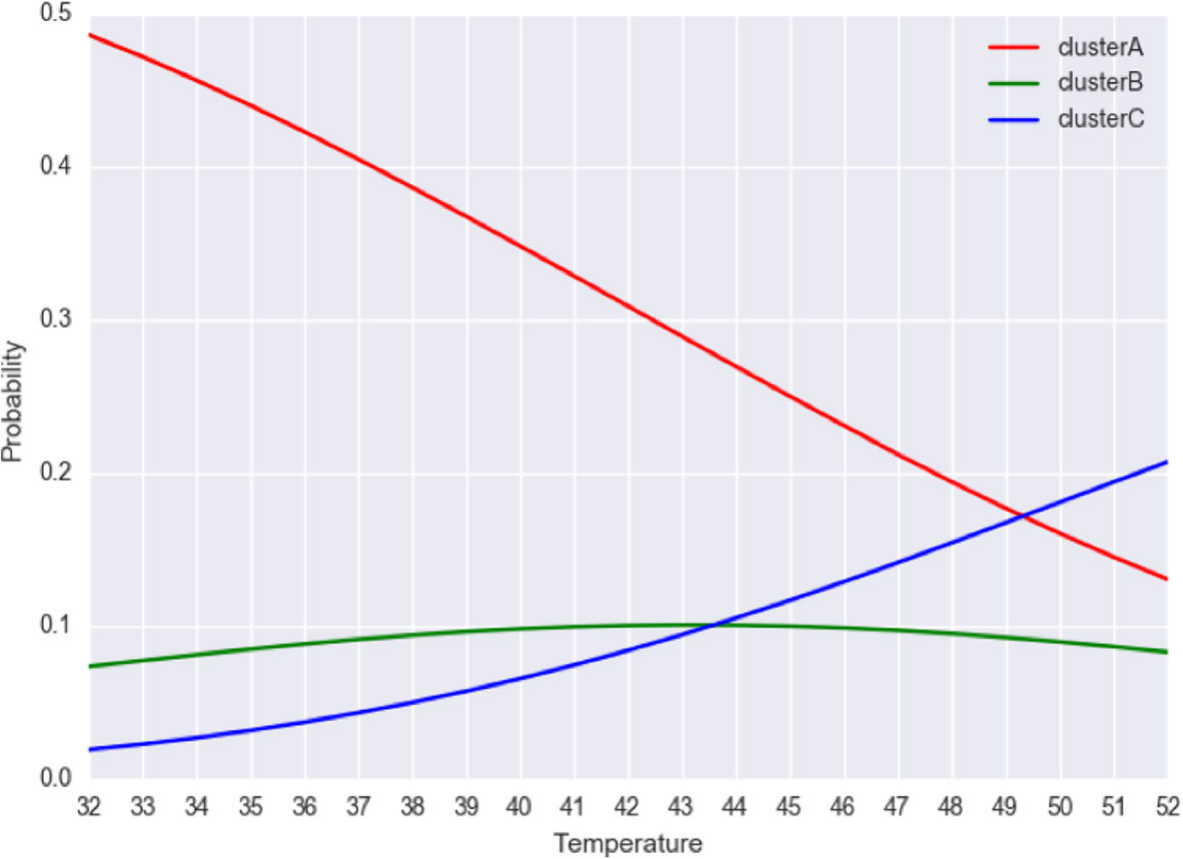
Probabilities of three structural clusters of ROSE element thermometer on different temperatures

Figure 4 shows the secondary structure probability distribution over Hamming distance of the ROSE element thermometer, where three major peaks for potential structural clusters are observed. We classified the three potential structural clusters A, B and C by Hamming distances, [0,10], [11,34] and [35,40]. The abundance of the clusters in their probabilities along the changing temperature is shown in Fig. 5.

**Fig. 6:**
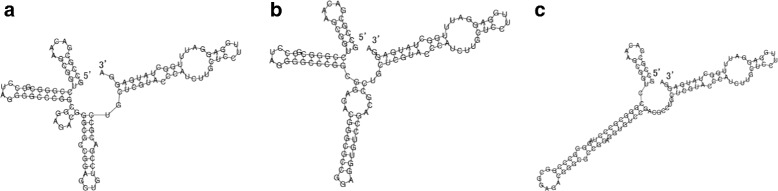
*γ*-centroid structures (*γ*=1) of three clusters of ROSE element thermometer. Structures **a**, **b** and **c** correspond to those obtained from the Hamming distance ranges of [0,10], [11,34] and [35,40]

**Fig. 7:**
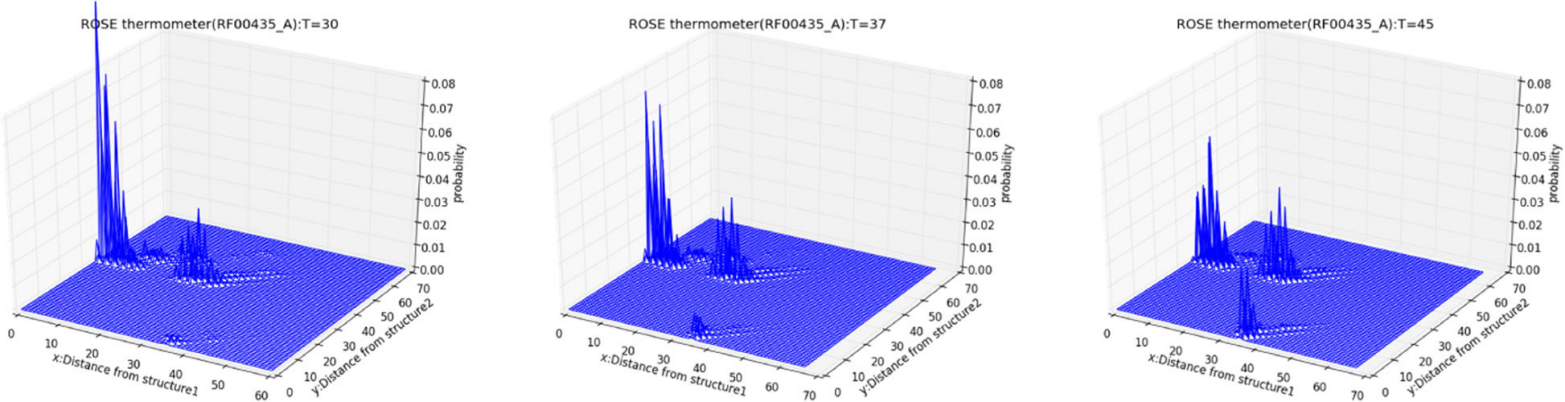
Distribution over Hamming distance (2D) of ROSE element thermometer, under the temperatures, 30 °C (left), (b) 37 °C (middle) and 45 °C (right). The first reference structure was obtained using the *γ*-centroid estimator (*γ*=1) with the Hamming distance [0,10] (Structure A, in Fig. 6). The second reference structure is obtained similarly from the Hamming distance [35,40] (Structure C, in Fig. 6)

We then calculate the base-pairing probabilities over Hamming distance by RintW, and *γ*-centroid estimators (*γ*=1) for the three clusters. The esimated *γ*-centroid structures are shown in Fig. 6. Using the *γ*-centroid estimators of the first and the third peak as the reference structures, the secondary structure probability distribution over Hamming distances (2D) were calculated under three different temperatures (Fig. 7), which shows the change of the probability landscape depending on the temperature. The change of probabilities of the three clusters depending on the temperature is shown in Fig. 5. It can be observed that cluster A (Hamming distance *d*∈[0,10]) is dominant in low temperature and that cluster C (*d*∈[35,40])become stronger in high temperature.

## Discussion

It is known that the functions of RNAs are closely correlated to their secondary structures, but limited reliability of secondary structure predictions have been preventing effective functional analyses. There are many tools for secondary structure predictions, but any point estimate of secondary structure has very small probability.

If the probabilities are concentrated in a cluster, it is possible to estimate an appropriate representative structure in the cluster. The Boltzmann distribution, however, often has multiple clusters of the concentration. Such concentrations may reflect of the essential alternative structures associated to switching functions, which are observed in several functional RNAs such as ribo-switches and thermometers.

There is no convenient tool to capture such essential alternative structures, though some tools can predict sub-optimal structures [7]. In this paper, we demonstrated that potential clusters of essential alternative secondary structures can be found by 1D analysis over Hamming distance. After detecting the candidates of clusters and defining the range of Hamming distance for each, the decomposed base-pairing probability matrix is computed by RintW. By using the representative structure calculated by posterior decoding of the base-pairing probability matrix, the 2D probability distributions (Figs. 7 and 3 bottom) are obtained by RintD [12]. If necessary, we can re-define the structural cluster based on the 2D distribution to re-calculate the decomposition of base-pairing probabilities.

In case of the ROSE element thermometer, the detection of the essential structural cluster from Fig. 4 was straightforward. The detection of the clusters, however, is not always easy. In the case of the Lysine riboswitch (Fig. 3), it was difficult to detect the peaks from simple 1D probability distribution, and log probability was informative. Such low probability region may have been ignored previously to overlook functionally relevant structures. It will be our future work to develop a method to find functionally important structural clusters by detailed analysis of the distribution, and also by better distance measure of the secondary structures than the Hamming distance.

## Conclusion

In this paper, we presented a new method to detect the essential alternative secondary structures from RNA sequences by decomposing the base-pairing probability matrix. In order to calculate the decomposition, we have developed RintW, which efficiently calculates the inside/outside partition functions over Hamming distance and the base-pairing probabilities. Those calculations utilized dynamic programming mapped to polynomials and application of discrete Fourier transformation. By applying the method to the Lysine riboswitch and ROSE element RNA thermometer, potential alternative structural clusters, which may reflect their change in conformation, were observed. In the case of the ROSE element RNA thermometer, it was shown that changing temperature affected abundance of the clusters in their probabilities. Those results have shown that our method have a strong potential to analyze functional RNAs which have essential alternative structures.
